# Plant recognition by AI: Deep neural nets, transformers, and kNN in deep embeddings

**DOI:** 10.3389/fpls.2022.787527

**Published:** 2022-09-27

**Authors:** Lukáš Picek, Milan Šulc, Yash Patel, Jiří Matas

**Affiliations:** ^1^Department of Cybernetics, Faculty of Applied Sciences, University of West Bohemia, Pilsen, Czechia; ^2^Visual Recognition Group, Department of Cybernetics, Faculty of Electrical Engineering, Czech Technical University in Prague, Prague, Czechia

**Keywords:** plant, species, classification, recognition, machine learning, computer vision, species recognition, fine-grained

## Abstract

The article reviews and benchmarks machine learning methods for automatic image-based plant species recognition and proposes a novel retrieval-based method for recognition by nearest neighbor classification in a deep embedding space. The image retrieval method relies on a model trained *via* the Recall@k surrogate loss. State-of-the-art approaches to image classification, based on Convolutional Neural Networks (CNN) and Vision Transformers (ViT), are benchmarked and compared with the proposed image retrieval-based method. The impact of performance-enhancing techniques, e.g., class prior adaptation, image augmentations, learning rate scheduling, and loss functions, is studied. The evaluation is carried out on the PlantCLEF 2017, the ExpertLifeCLEF 2018, and the iNaturalist 2018 Datasets—the largest publicly available datasets for plant recognition. The evaluation of CNN and ViT classifiers shows a gradual improvement in classification accuracy. The current state-of-the-art Vision Transformer model, ViT-Large/16, achieves 91.15% and 83.54% accuracy on the PlantCLEF 2017 and ExpertLifeCLEF 2018 test sets, respectively; the best CNN model (ResNeSt-269e) error rate dropped by 22.91% and 28.34%. Apart from that, additional tricks increased the performance for the ViT-Base/32 by 3.72% on ExpertLifeCLEF 2018 and by 4.67% on PlantCLEF 2017. The retrieval approach achieved superior performance in all measured scenarios with accuracy margins of 0.28%, 4.13%, and 10.25% on ExpertLifeCLEF 2018, PlantCLEF 2017, and iNat2018–Plantae, respectively.

## 1. Introduction

Accurate species identification is essential for most ecologically motivated studies, in the pharmaceutical industry, agriculture, and conservation. In the case of Flora—with more than 400,000 species and high inter-species similarities—correct species determination requires a high level of expertise. An identification process using dichotomous keys may take days, even for specialists, especially in locations with high biodiversity, and it is exceedingly difficult for non-scientists (Belhumeur et al., 2008). To overcome that issue, Gaston and O'Neill (2004) proposed to use a computer vision based search engine to partially assist with plant identification and consequentially speed up the identification process. Since then, we have witnessed an increased research interest in plant species identification using computer vision and machine learning (Wu et al., 2006, 2007; Prasad et al., 2011; Priya et al., 2012; Caglayan et al., 2013; Munisami et al., 2015), especially following the advances in deep learning (Ghazi et al., 2017; Bonnet et al., 2018; Lee et al., 2018; Šulc et al., 2018; Wäldchen and Mäder, 2018; Picek et al., 2019).

The overall performance of automatic fine-grained image classifiers has improved considerably over the last decade with the development of deep neural networks, mostly Convolutional Neural Networks (CNNs). We refer readers unfamiliar with the principles of deep learning and CNNs to the book by Goodfellow et al. (2016). The success of deep learning models trained with full supervision is typically conditioned by the existence of large databases of annotated images. For plant recognition, such large-scale data are available, thanks to citizen-science and open-data initiatives such as Encyclopedia of Life (EoL), Pl@ntNet, and the Global Biodiversity Information Facility (GBIF). This allowed building challenging datasets for fine-grained classification training and evaluation, e.g., in PlantCLEF (Goëau et al., 2016, 2017, 2018, 2020, 2021), LifeCLEF (Joly et al., 2018, 2019, 2020, 2021), iNaturalist (Van Horn et al., 2018), and Pl@ntNet (Garcin et al., 2021).

**Figure 1:**
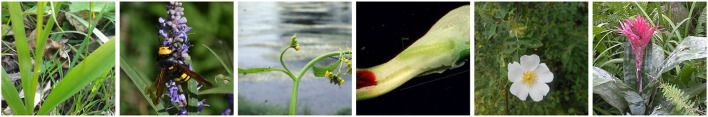
“In the wild” photograph samples—PlantCLEF datasets. Images by soyoban, Liliane Roubaudi, Hugo Santacreu, Sarah Dechamps, Richard Gautier, Heinz Gass, Alain Bigou, Jean-Michel Launay, and Jose Luis Romero.

This article deals with automatic image-based plant species identification “*in the wild”*, thus dealing with: (i) Different scales: Plant species can be observed from various angles and distances. (ii) Intra-class differences: Plant organs—leaf, fruit, bark, etc.—look very distinct. (iii) Inter-class similarities: The same organ of different species might look very similar. (iv) Background and Clutter: Other species are present behind or around the observed sample, and many more. Identification of plants from images is a fine-grained classification problem, due to the high number of classes^1^, high intra-class variance, and small inter-class differences. Šulc and Matas (2017) showed that constrained plant identification tasks, such as recognition of scanned leaves, can be solved with a high level of classification accuracy (± 99%). Yet the “*in the wild”* scenario, with an unspecified view or organ type, natural background, possible clutter in the scene, etc., remains challenging even for state-of-the-art deep learning methods. For “In the wild” photograph samples, refer to Figure 1.

First, is the standard approach, where fine-grained recognition is posed as closed-set classification; the learning involves minimization of cross-entropy loss. Second, a retrieval-based approach, which is very competitive, achieves superior in comparable conditions. Here, the training involves learning an embedding where the metric space leads to high recall in the retrieval task. Formulating fine-grained recognition as retrieval has clear advantages—besides providing ranked class predictions, it recovers relevant nearest-neighbor labeled samples. The retrieved nearest neighbors provide explainability to the deep network and can be visually checked by an expert. Moreover, the user may inspect specific information, e.g., about location and date of collection, to further reduce decision uncertainty. Besides, the retrieval approach naturally supports open-set recognition problems, i.e., the ability to extend or modify the set of recognized classes after the training stage. The set of classes may change, e.g., as a result of modifications to biological taxonomy. New classes are introduced simply by adding training images with the new label, whereas in the standard approach, the classification head needs re-training. On the negative side, the retrieval approach requires, on top of running the deep net to extract the embedding, to execute the nearest neighbor search efficiently, increasing the overall complexity of the fine-grained recognition system.

Section 4 discusses techniques that can noticeably improve the performance of any vision-based species recognition system. The techniques are diverse and attend to different problems. The prior shift in the datasets, i.e., the difference between the training and test data class distribution, is a significant and omnipresent phenomenon. We test existing prior shift adaptation methods and their impact on classification accuracy. Class prior adaptation equips the system with the ability to reflect the change of prior probability of observing a specimen of a given species over time and location. Image augmentations make the system robust to acquisition conditions that, in some applications, e.g., plant recognition, are far from the lab setting. Finally, technical aspects related to training of the deep nets, such as learning rate schedule, loss functions and the impact of the noisy data, on classification performance, are discussed.

The performance evaluation part of the article builds on our winning submissions to PlantCLEF (Picek et al., 2019; Sulc and Matas, 2019) and extends a workshop article (Šulc et al., 2018) and a PhD thesis (Šulc, 2020). It substantially extends the experiments by including recent state-of-the-art methods for image classification: Convolutional Neural Networks (CNNs) (Xie et al., 2017; Hu et al., 2018; Zhang et al., 2020; Tan and Le, 2021), Vision Transformers (ViTs) (Dosovitskiy et al., 2021), and an interpretable image retrieval approach (Patel et al., 2021).

## 2. Related work

This chapter reviews existing methods, systems, and applications for plant species recognition: leaf or bark recognition and “*in the wild*” plant species recognition.

### 2.1. Leaf and bark recognition

Leaf and bark recognition was the only application before deep learning where automatic plant species identification allowed to reliably tackle complex species recognition tasks. Most techniques were based on two steps: (i) descriptor extraction, often based on combining different hand-crafted features such as shape, color, or local descriptors (SIFT, SURF, ORB, etc.), and (ii) classical. classifiers such as k-Nearest Neighbor (Munisami et al., 2015), Random Forest (Caglayan et al., 2013), SVM (Prasad et al., 2011; Priya et al., 2012), and early adoptions of neural networks (Wu et al., 2006, 2007). The generalization capability of these methods was limited, and so was the applicability—e.g., most leaf recognition methods relied on the shape of scanned leaves; thus, the usability in the “in the wild” scenario was limited since the uniform background was required.

### 2.2. Flora recognition in the wild

The continuous progress in automatic plant species recognition “*in the wild*” has been strongly driven by the efforts of the LifeCLEF research platform. Established in 2014, the LifeCLEF helps track progress and allows reliable evaluation of novel methods. In particular, the annual PlantCLEF challenges are an immense source of plant species datasets tailored to develop and evaluate automatic plant species recognition methods.

Following the findings of the LifeCLEF challenges (Joly et al., 2018, 2019, 2020, 2021), AI-based identification of the world flora has improved significantly over the last 5 years, and it reached similar performance as human experts for common (Šulc et al., 2018) as well as for rare species (Picek et al., 2019). Ensembles of CNN models were able to recognize 10,000 plant species from Europe and North America and 10,000 from the Guiana shield and the Amazonia with approximately 90 and 40% accuracy, respectively.

Overall, there are few methods for plant recognition “in the wild”; thus, we overview relevant methods for general fine-grained recognition. Wu et al. (2019) developed a Taxonomic Loss that sums up loss functions calculated from different taxonomy ranks, e.g., species, genus, and family. Cui et al. (2018) studied domain-specific transfer learning from large-scale datasets to domain-specific fine-grained datasets. Zheng et al. (2019) propose the Trilinear Attention Sampling Network that generates attention maps by modeling the inter-channel relationships, highlights attended parts with high resolution and distills part features into an object-level feature. Keaton et al. (2021) utilized object detection as a form of attention with a bottom-up approach to detect plant organs and combine the predictions from organ-specific classifiers. Malik et al. (2021) used a standard ensemble-based approach utilizing Inception, MobileNet and ResNet CNN architectures.

Several interesting approaches emerged in connection with the annual PlantCLEF workshops. In PlantCLEF 2017, the best performing submission competition with an accuracy of 88.5% was developed by Lasseck (2017). The underlying method is based on 12 models derived from 3 architectures—GoogLeNet, ResNet-152, and ResNeXt-101-64x4d. All models were fine-tuned from the ImageNet-1k checkpoints utilizing various augmentation techniques, e.g., random cropping, horizontal flipping, variations of saturation and lightness, and rotation. While testing, 5 crops for all observation images are predicted with all models and averaged. In the PlantCLEF 2018, the best performing submission (Sulc and Matas, 2019) was based on two architectures—Inception-ResNet-v2 and Inception-v4 (Szegedy et al., 2017)—and their ensembles and achieved an accuracy of 88.4%. The TensorFlow-Slim API was used to adjust and fine-tune the networks from the publicly available ImageNet-1k pre-trained checkpoints. All networks shared the following optimizer settings: RMSprop with momentum and decay set to 0.9, initial learning rate 0.01, and exponential learning rate decay factor 0.4. Batch size, input resolution, and random crop area range were set differently for each network. For the used values please refer to the original article (Sulc and Matas, 2019). The following image pre-processing was used for training: Random crop, with aspect ratio range (0.75, 1.33) and with various area ranges, Random left-right flip, and Brightness and Saturation distortion. At test-time, 14 predictions per image are generated by using 7 crops and their mirrored versions: full image, central crop covering 80% of the original image dimensions, central crop covering 60% of the original image dimensions, and 4 corner crops covering 60% of the original image dimensions. The significant improvement in accuracy was achieved by using running averages of the trained variables instead of the values from the last training step. This is important especially if the noisy labels are present in the training set where mini-batches with noisy samples may produce large gradients pointing outside of the local optima. The use of the Polyak averaging (Polyak and Juditsky, 1992) resulted in a more stable version of the training variables.

## 3. Datasets

This section overviews datasets suitable for plant recognition “*in the wild*” which, unlike other plant species datasets, contain images of various plant body parts observed in an open world. Such datasets are unique with high inter-class similarities—bark of one species is similar to the bark of another species—and high intra-class differences—the bark, flower, and fruit of one species are visually distinct. Currently, datasets with large species diversity and a sufficient number of samples to train a reliable machine learning model are available. The most significant providers of those datasets—iNaturalist, Pl@ntNet, EoL, LifeCLEF—are closely connected to citizen-science platforms, thus their data originate from thousands of users, and are captured on various devices, observed under different conditions, and submitted from many countries. The most influential datasets are described below and their main characteristics are summarized in Table 1.

**Table 1:** Datasets for plant recognition; “*in the wild*” scenario.

		**Number of images in**		
**Dataset**	**Species**	**Training**	**Validation**	**Test**
Pl@ntNet-300K	1,081	243,916	31,118	31,112
iNaturalist 2017^†^	2,101	158,407	38,206	×
iNaturalist 2018^†^	2,917	118,800	8,751	×
iNaturalist 2021^†^	4,271	1,148,702	42,710	×
PlantCLEF 2016	1,000	113,205	×	2,583
PlantCLEF 2017^‡^	10,000	320,544	×	25,170
ExpertLifeCLEF 2018^‡^	10,000	320,544	×	6,892
PlantCLEF 2019	10,000	434,251	×	2,974

Species from the *Plantae* kingdom marked^†^, data with “*trusted*”, i.e., human verified, labels marked^‡^.

For the experimental evaluation in this article, we used iNaturalist 2018^†^, PlantCLEF 2017^‡^, and ExpertLifeCLEF 2018^‡^, as they offer a sufficient number of species and test samples while keeping the training set size and, thus, computational demands reasonably low.

### 3.1. LifeCLEF—PlantCLEF

**Figure 2:**
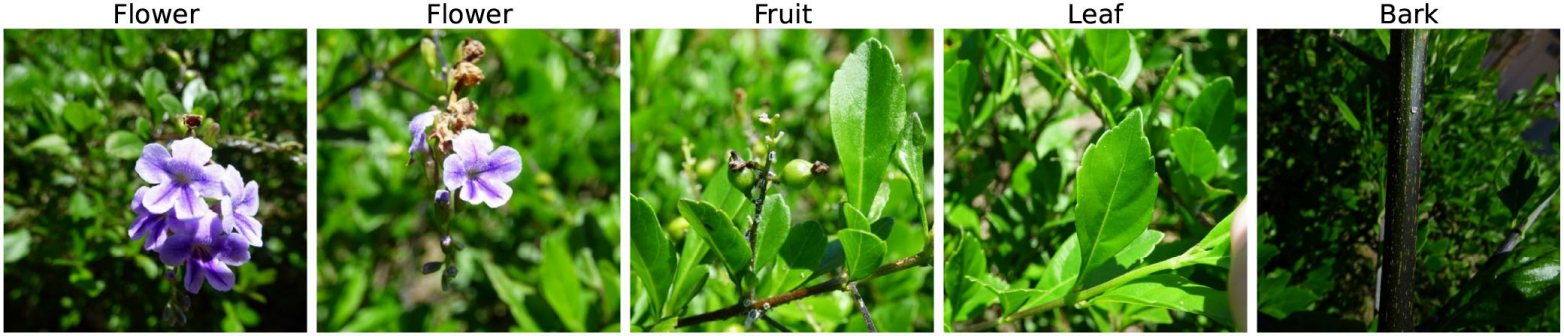
A PlantCLEF observation—images of different plant parts. Images by Hugo Santacreu.

The annual LifeCLEF—PlantCLEF identification challenge is an important source of data for plant recognition. Since 2017 the PlantCLEF challenges present the following classification problem: For each plant observations consisting of one or more images of the same specimen, predict the species. Example images from one observation are visualized in Figure 2. The PlantCLEF datasets are mainly intended for benchmarking machine-learning-based algorithms for plant recognition, thus are briefly described below.

**The PlantCLEF 2016** dataset (Goëau et al., 2016) comprises 1,13,205 training images belonging to 41,794 observations of 1,000 plant species from France and neighboring countries. Every image is annotated with a plant organ label, i.e., flower, leaf, fruit, stem, branch, and whole plant. A small fraction has GPS coordinates. The test set contains 2,583 images. As in all PlantCLEF challenges, no predefined validation set was provided.

**The PlantCLEF 2017** challenge dataset (Goëau et al., 2017) includes 3,20,544 images from the Encyclopedia of Life with trusted labels, and noisy web data crawled with Bing and Google search engines (~1.15M images). The dataset covers 10,000 plant species—mainly from North America and Europe—representing the biggest plant species identification dataset in the number of classes. The test set contains 25,170 images (17,868 observations).

**The ExperLifeCLEF 2018** training dataset (Goëau et al., 2018) differs from the PlantCLEF 2017 dataset only in the test set. The test set contains 6,892 images (2,072 observations) covering species mainly from Western Europe and North America. In addition, selected endangered species, and cultivated and ornamental plant species were added.

**The PlantCLEF2019** dataset (Goëau et al., 2019) contains 434,251 images that belong to 10,000 rare species from the Guiana shield and the Amazon rain forest.The images originate from EoL and Google/Bing search engines; the majority have the “*noisy*” labels. The test set is composed of 742 plant observations (2,974 images) collected and identified by five experts on tropical flora.

### 3.2. iNaturalist

iNaturalist is a crowd-based citizen-science platform allowing citizens and experts to upload, annotate and categorize species of the world. iNaturalist has a wide geographic and taxonomic coverage—more than 343 thousand species with approximately 97 million observations. The annual iNaturalist competition datasets that include a significant number of plant species are described below.

**iNaturalist 2017**: The iNaturalist 2017 dataset (Van Horn et al., 2018) contains 2,101 plant species, with 1,58,407 training and 38,206 validation images that have been collected and verified by multiple independent users. The dataset features many visually similar species that have been captured worldwide and under various conditions. As labels for the test set were not provided, it is impossible to specify how many plant species are contained.

**iNaturalist 2018**: The iNaturalist Challenge 2018 dataset includes 2,917 plant species, with 118,800 training and 8,751 validation images acquired the same way as in the previous year. Additionally, complete taxonomy information was given for all images. Test labels were not provided.

**iNaturalist 2021**: The iNaturalist Challenge 2021 dataset with 1,148,702 training and 42,710 validation images is the most extensive dataset considering the number of images—the number of plant species was increased to 4,271. Test labels were not provided as in all iNaturalist Challenge datasets.

### 3.3. Pl@ntNet-300K

The Pl@ntNet-300K dataset Garcin et al. (2021) is built from the database of the Pl@ntNet citizen observatory and includes 1,081 species and 306,146 images. The dataset exhibits a long-tailed class imbalance, where 20% of the most common species provide 89% of the images. Provided validation and test sets include 31,118 and 31,112 images, respectively.

## 4. Methods

This section is divided into three parts. First, the pipeline for automatic Plant Recognition by the standard Image Classification pipeline is described. Second, an alternative and novel approach to Plant Recognition *via* kNN classification in deep embedding space is proposed and described. Finally, a range of methods and techniques that increase classification performance are introduced.

### 4.1. Deep neural network classifiers

Plant species recognition can be easily automated through the standard image classification approach, where a Deep Neural Network (DNN) serves as a deep feature extractor and a fully convolutional neural network as a classifier. Image representations learned by deep neural networks provide significantly better results than handcrafted features. Furthermore, DNNs are data-driven and require no effort or expertise for feature selection as they automatically learn discriminative features for every task. In addition, the automatically learned features are represented hierarchically on multiple levels. Having such deep features is a strong advantage over traditional approaches.

Currently, many DNN architectures are widely used; thus, a broad range of Convolutional Neural Networks and Transformer-based architectures are evaluated to test the classification capabilities for different feature extractor architectures. The ResNet-50 (He et al., 2016), Inception-v4, and Inception-ResNet-v2 (Szegedy et al., 2017) are chosen as baselines as they are commonly used in related study. We add the following novel and state-of-the-art architectures:

**SE-ResNeXt-101:** Extends the ResNet deep residual blocks by adding the *NeXt* dimension, called Cardinality (Xie et al., 2017), and Squeeze and Excite blocks that adaptively re-calibrates channel-wise feature responses by explicitly modeling inter-dependencies between channels (Hu et al., 2018).

**ResNeSt-269e:** Applies channel-wise attention to different parts of the architecture to leverage and allow the cross-feature interactions and learning of the more diverse representations. (Zhang et al., 2020).

**EfficientNetV2-S:** Similarly to the first EfficientNet generation, the EfficientNet-v2 architectures are developed by a combination of training-aware architecture search and scaling, to jointly optimize training speed and parameter efficiency (Tan and Le, 2021). Newly, the models: (i) were searched from the space enriched with Fused-MBConv, and (ii) the last stride-1 stage in the original EfficientNet was removed.

**Vision Transformers:** Unlike CNN, the Vision Transformer (ViT) (Dosovitskiy et al., 2021) does not use convolutions but interprets an image as a sequence of patches and processes it by a standard Transformer encoder used primarily for natural language processing (Vaswani et al., 2017). Compared to state-of-the-art convolutional networks, selected ViT architectures demonstrated excellent performance in fine-grained image classification (Picek et al., 2022).

#### 4.1.1. Training strategy

**Figure 3:**
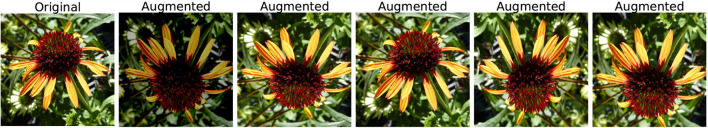
Image augmentations—Horizontal and vertical flip, small brightness/contrast adjustments, and 80–100% crops—used while training the deep neural network classifier. Image by Zoya Akulova.

All NN architectures were initialized from publicly available ImageNet-1k or ImageNet-21k pre-trained checkpoints (Wightman, 2019) and further fine-tuned for 100 epochs. Mini-batch gradients were accumulated to reach an effective size of 128 for all the architectures—most of the time, 4 batches of size 32 are accumulated. SGD with momentum (0.9) was used as an optimizer with a custom learning rate (LR) schedule—Reduce LR to a fraction of 0.9 if validation loss does not decrease for 2 epochs. The loss was calculated as Softmax Cross Entropy. While training, we employ a few data augmentation techniques from the Albumentations library (Buslaev et al., 2020). A sample image and its augmented variations are shown in Figure 3. Augmentation methods, their description, and specified non-default parameters are:

- *RandomResizedCrop*: creates a random resized crop with a scale of 0.8 − 1.0.
- *HorizontalFlip*: randomly (50% probability) flips the image horizontally.
- *VerticalFlip*: randomly (50% probability) flips the image vertically.
- *RandomBrightnessContrast*: changes contrast and brightness by a random factor in a range −0.2 − 0.2 with 20% probability.

All images were: resized to match the pre-trained model input size of 224 × 224 or 384 × 384, re-scaled from 0 − 255 to 0 − 1, and normalized by mean (0.5) and std (0.5) values in each channel.

#### 4.1.2. Test-time

At the test time, all images are resized to the appropriate size, i.e., 224 × 224 or 384 × 384, and normalized as in training. Next, all observation images are feed-forward and class predictions are combined. The study about different methods for prediction combinations is included in Section 5.3. The classification performance for all selected models is evaluated on both resolutions—224 × 224 and 384 × 384—and two different test sets—PlantCLEF 2017 and ExpertLifeCLEF 2018.

### 4.2. Plant recognition *via* kNN classification in deep embedding space

Fine-grained recognition of plant species can be alternatively solved *via* the k-Nearest Neighbors algorithm (kNN) in an embedding space where the samples from the same semantic class are grouped together, and the samples from different classes are far apart. Recent study by Touvron et al. (2021); Khosla et al. (2020) have shown such a recognition technique to outperform standard cross entropy based training. For training of such an embedding, we use the current state-of-the-art image retrieval method Patel et al. (2021), where a deep neural network is trained on a surrogate loss—Recall@k. The notations and methodology for the retrieval approach are described below.

#### 4.2.1. Notations

For a query example *q* ∈ *X*, the objective of a retrieval model is to obtain semantically similar samples from a collection Ω ⊂ *X*, also known as database, where *X* is the space of all images. The database is divided into two subsets based on the positive or negative samples to the query *q*. These subsets are denoted by *P*_*q*_ and *N*_*q*_, respectively, such that Ω = *P*_*q*_ ∪ *N*_*q*_. For the query *q*, all database samples are ranked based on a similarity score, with the goal to rank positives before negatives.

#### 4.2.2. Deep embedding

Image embedding, a learned vector representation of an image, is generated by function $f_{\theta }:X\to R^{d}$. Function *f*_θ_ is a deep neural network, either a ResNet-50 or a Vision Transformer in this article, mapping input images to an *L*_2_-normalized *d*-dimensional embedding. Embedding for image *x* is denoted by ***x*** = *f*_θ_(*x*). Parameters θ of the network are learned during the training using Recall@k surrogate loss. The similarity score between a query *q* and a database image *x* is computed by the dot product of the corresponding embeddings and is denoted by *s*(*q, x*) = ***q***^*T*^***x***, also denoted as *s*_*qx*_.

#### 4.2.3. Recall@k surrogate loss

The Recall@k Surrogate loss is a differentiable approximation of the Recall@k evaluation metric. For a query *q*, the Recall@k metric is the ratio of positive (relevant) samples in top-k retrieved samples to the total number of positive samples in the database, given by |*P*_*q*_|. The metric focuses only on top-k ranked samples and is one of the standard metrics to evaluate retrieval benchmarks. Recall@k cannot be directly used as a loss function. It requires two non-differentiable operations: ranking the database samples and counting the number of positives that appear in top-k. The subsequent text presents Recall@k expressed mathematically, non-differentiability, and the differentiable approximation as proposed by Patel et al. (2021).

Patel et al. (2021) denotes Recall@k by $R_{\text{Ω}}^{k}(q)$ when computed for query *q* and database Ω and expresses it mathematically in terms of ranks of samples in the database:


(1) $$\begin{array}{l} R_{\Omega }^{k}(q)=\frac{\sum_{x\in P_{q}}H(k-r_{\Omega }(q,x))}{\left|P_{q}\right|}, \end{array}$$

where the rank of sample *x* is denoted by *r*_Ω_(*q, x*), which depends on the query sample *q* and the database Ω. *H*(.) is the Heaviside step function, which is 0 for negative values and otherwise 1. The rank *r*_Ω_(*q, x*) of sample *x* is computed according to the similarity score, and it can be expressed mathematically as:


(2) $$\begin{array}{l} r_{\text{Ω}}\left(q,x\right)=1+\underset{z\in \text{Ω},z\neq x}{\sum }H\left(s_{qz}-s_{qx}\right), \end{array}$$

where *H*(.) is also the Heaviside step function applied on the difference of similarity scores. Therefore, Recall@k from Equation (1) can also be directly expressed as a function of similarity scores as:


(3) $$\begin{array}{l} R_{\text{Ω}}^{k}(q)=\frac{\sum_{x\in P_{q}}H(k-1-\sum_{z\in \text{Ω},z\neq x}H(s_{qz}-s_{qx}))}{\left|P_{q}\right|}. \end{array}$$

The computation of Recall@k in Equation (3) involves the use of two Heaviside step functions, one to obtain the rank and the other to count the positives in top-k retrieved samples. The gradient of the Heaviside step function is a Dirac delta function. Hence, direct optimization of recall with back-propagation is not feasible. Patel et al. (2021) provide a smooth approximation of the Heaviside step function by the logistic function, a sigmoid function σ_τ_:*R*→*R* controlled by temperature τ:


(4) $$\begin{array}{l} \sigma_{\tau }\left(u\right)=\frac{1}{1+e^{-\frac{u}{\tau }}}, \end{array}$$

Replacing the two Heaviside step functions with the sigmoid functions of appropriate temperatures, a smooth approximation of Recall@k can be expressed as:


(5) $$\begin{array}{l} {\tilde{R}}_{\Omega }^{k}(q)=\frac{\sum_{x\in P_{q}}\sigma_{\tau_{1}}(k-1-\sum_{\begin{array}{c} z\in \Omega \\ z\neq x \end{array}}\sigma_{\tau_{2}}(s_{qz}-s_{qx}))}{\left|P_{q}\right|}, \end{array}$$

The Recall@k Surrogate loss from Equation (5) is differentiable and is used for training the parameters θ of the deep embedding model. In practice, the Recall@k Surrogate loss is re-scaled to have values between 0 and 1, by dividing it by min(*k*, |*P*_*q*_|) instead of |*P*_*q*_|, and by clipping the values larger than *k* in the numerator. The single-query loss to be minimized in a mini-batch *B*, with size |*B*|, and query *q*∈*B* is given by:


(6) $$\begin{array}{l} L^{k}\left(q\right)=1-{\tilde{R}}_{B\backslash q}^{k}\left(q\right). \end{array}$$

The final loss is computed by averaging the loss across multiple values of *k* as:


(7) $$\begin{array}{l} L^{K}\left(q\right)=\frac{1}{\left|K\right|}\underset{k\in K}{\sum }L^{k}\left(q\right). \end{array}$$

In practice, we use following values *K* = {1, 2, 4, 8, 16}. All examples in the mini-batch are used as queries, and the average loss over all queries is minimized during the training.

#### 4.2.4. Training

The training is set up for 100 epochs using an AdamW optimizer (Loshchilov and Hutter, 2019) with an initial learning rate of 0.0001, which decreases by a factor of 0.3 using a step decay. For data augmentation, images are resized to 256 × 256, and a random crop of 224 × 224 is taken, followed by a random horizontal flip with a probability of 0.5 and normalization with mean and SD. The mini-batch is constructed *via* class-balanced sampling with 4 samples per class and a large batch size of 4, 000 is used. Two feed-forward passes (Patel et al., 2021) are accumulated to create a larger batch size to address the GPU hardware demands. The first feed-forward pass is performed on the batch with 4, 000 samples in chunks of 200 samples at a time. All embedding vectors are stored while the intermediate features are discarded from the GPU memory. Using the embedding vectors and the ground truth labels, the loss (Equation 7) and the gradients for each sample with respect to the embedding vectors are calculated. Finally, a second feed-forward is performed, also in the chunks of 200 samples at a time, allowing the propagation of the gradients through the deep embedding model for the current chunk of 200 samples. At the end of the second feed-forward stage, the model's weights are updated.

#### 4.2.5. Test-time

At inference, the test image is resized to 256 × 256, and a central crop of 224 × 224 with normalization is the input to the deep embedding model. A feed-forward pass is performed through all the training and testing samples, and the embedding vectors are stored. Each test sample is treated as a query for retrieval, and the ten closest samples from the training set are obtained. A majority vote determines the semantic class of the test sample.

### 4.3. Class prior estimation

Commonly in Machine Learning, the class prior probabilities are the same for the training data and test data. However, plant species distributions change dramatically based on various aspects, i.e., seasonality, geographic location, weather, the hour in a day, etc. The problem of adjusting CNN outputs to the change in class prior probabilities was discussed in Sulc and Matas (2019), where it was proposed to recompute the posterior probabilities (predictions) *p*(*c*_*k*_|**x**_*i*_) by Equation (8).


(8) $$\begin{array}{l} p_{e}(c_{k}|x_{i})=p(c_{k}|x_{i})\frac{p_{e}(c_{k})p(x_{i})}{p(c_{k})p_{e}(x_{i})}=\frac{p(c_{k}|x_{i})\frac{p_{e}(c_{k})}{p(c_{k})}}{\sum_{j=1}^{K}p(c_{j}|x_{i})\frac{p_{e}(c_{j})}{p(c_{j})}}\;\\ \;\propto p(c_{k}|x_{i})\frac{p_{e}(c_{k})}{p(c_{k})}, \end{array}$$

The subscript *e* denotes probabilities on the evaluation/test set. The posterior probabilities *p*(*c*_*k*_|**x**_*i*_) are estimated by the Convolutional Neural Network outputs since it was trained with the cross-entropy loss. For class priors *p*(*c*_*k*_), we have an empirical observation—the class frequency in the training set. The evaluation and test set priors *p*_*e*_(*c*_*k*_) are, however, unknown. To evaluate the impact of changing class priors, we compare three existing prior estimation algorithms—the Expectation–maximization algorithm (EM) of Saerens et al. (2002) and the recently proposed CM-L and SCM-L methods of Sipka et al. (2022).

#### 4.3.1. EM—expectation maximization

In our ExpertLifeCLEF 2018 challenge submissions, we followed the proposition from Sulc and Matas (2019) to use an EM algorithm of Saerens et al. (2002) for the estimation of test set priors by maximization of the likelihood of the test observations. The E and M step are described by Equation (9), where the super-scripts (*s*) or (*s* + 1) denote the step of the EM algorithm.


(9) $$\begin{array}{l} \begin{array}{cc} p_{e}^{\left(s\right)}\left(c_{k}|x_{i}\right) & =\frac{p\left(c_{k}|x_{i}\right)\frac{p_{e}^{\left(s\right)}\left(c_{k}\right)}{p\left(c_{k}\right)}}{\overset{K}{\underset{j=1}{\sum }}p\left(c_{j}|x_{i}\right)\frac{p_{e}^{\left(s\right)}\left(c_{j}\right)}{p\left(c_{j}\right)}},\\ p_{e}^{\left(s+1\right)}\left(c_{k}\right) & =\frac{1}{N}\overset{N}{\underset{i=1}{\sum }}p_{e}^{\left(s\right)}\left(c_{k}|x_{i}\right), \end{array} \end{array}$$

In our submissions, we estimated the class prior probabilities for the whole test set. However, one may also consider estimating different class priors for different locations, based on the GPS-coordinates of the observations. Moreover, as discussed by Sulc and Matas (2019), one may use this procedure even in the cases where the new test samples come sequentially.

#### 4.3.2. CM-L—confusion matrix based likelihood maximization

The prior estimate is based on maximizing the likelihood of the observed classifier decisions. The CM-L method uses the classifier's *confusion matrix* (CM) in the format **C**_*d*|*y*_, where the value in the *k*-th column and *i*-th row is the probability *p*(*D* = *i*|*Y* = *k*) of the classifier deciding for class *i* when the true class is *k*. The new class priors **P** are then estimated by maximizing the log-likelihood with the following objective:


(10a) $$\begin{array}{ll} \hat{P}= & \underset{\text{P}}{arg max}\ell \left(\text{P}\right)=\underset{\text{P}}{arg max}\overset{K}{\underset{k=1}{\sum }}n_{k}\log\left({\text{C}}_{k,:}\cdot \text{P}\right) \end{array}$$

(10b) $$\begin{array}{ll} \text{s.t.:} & \;\overset{K}{\underset{k=1}{\sum }}P_{k}=1;\;\forall k:P_{k}\geq 0, \end{array}$$

where *n*_*k*_ is the numbers of classifier's decisions for class *k* on test set and **C**_*k*, :_ is the *k*-th row of the confusion matrix.

The SCM-L method works analogically, but uses the so-called *soft confusion matrix* (SCM) ${\text{C}}_{d|y}^{\text{soft}}$ estimated from the classifier's soft predictions **f** as


(11) $$\begin{array}{l} {Ĉ}_{:,k}^{\text{soft}}=\frac{1}{N_{k}}\underset{{\text{x}}_{i}:y_{i}=k}{\sum }\text{f}\left({\text{x}}_{i}\right), \end{array}$$

where ${Ĉ}_{:,k}^{\text{soft}}$ denotes the *k*-th column of SCM. The probability $p_{E}^{\text{soft}}\left(D\right)$ can be estimated by averaging predictions **f**(**x**) over the test set.

## 5. Results

First, we compare the state-of-the-art Convolutional Neural Networks and Vision Transformers in Section 5.1. Second, we evaluate the image retrieval approach to classification and compare it with the standard classifiers in Section 5.2. Finally, additional techniques for performance improvements are evaluated in Section 5.3.

### 5.1. Image classification

#### 5.1.1. Combining several predictions per observation

LifeCLEF datasets include sets of images belonging to the same specimen observation. Typically, the images represent different organs of the specimen, e.g., flower, leaf, Such sets of images are connected by the ObservationID values provided in the metadata. The PlantCLEF 2017 test set contains 17,868 observations and 25,170 images. The ExpertLifeCLEF 2018 test set is smaller with 2,072 observations and 6,892 images. Plant species prediction based on multiple images is intuitive; it is inspired by the process used for years by botanists. Four simple approaches of per-image prediction combination are evaluated. Decide for the class with

- **Max softmax**: maximum posterior probability estimate—softmax—over all images, i.e., follow the most confident prediction,
- **Mean softmax**: maximum average (over images) estimated posterior probability,
- **Max logit**: maximum activation value (Logit) over all images.
- **Mean logits**: maximum average (over images) logit value.

The best results of species prediction combination was achieved by selecting the species with the maximum value of logit mean. For the single ViT-Base/32 model and image size of 224 × 224, the Mean logits approach outperformed the max softmax by 0.86% on PlantCLEF 2017 and 4.59% on ExpertLifeCLEF 2018. Overall, the accuracy is significantly higher for observations then for single images, in some cases increasing the accuracy by more then 20%. Full results are shown in Table 2.

**Table 2:** Classification accuracy on the PlantCLEF 2017 and the ExpertLifeCLEF 2018 datasets for different image prediction combination strategies.

**Architecture**	**Test set**	**Image-wise**	**Max Softmax**	**Mean Softmax**	**Max Logits**	**Mean Logits**
EfficientNetV2-S	2017	79.21	84.35	85.26	85.54	**85.75**
EfficientNetV2-S	2018	53.08	67.28	70.32	72.25	**74.13**
ViT-Base/32	2017	73.50	80.43	80.55	80.79	**81.29**
ViT-Base/32	2018	49.36	66.94	66.84	68.87	**71.53**

**Convolutional neural networks:** The comparison of the former and recent state-of-the-art CNN architectures on the PlantCLEF2017 and the ExpertLifeCLEF 2018 test sets shows similar behavior as on other fine-grained datasets (Wah et al., 2011; Van Horn et al., 2018; Picek et al., 2022). The best performing model on both datasets is EfficientNetV2-L with 77.03% accuracy on ExpertLifeCLEF 2018 and 88.52% accuracy on PlantCLEF 2017. Other deep networks including ResNeSt-269e and SE-ResNeXt-101 underperformend by a significant margin. The achieved scores are summarized in Table 3.

**Table 3:** Image classification accuracy for Deep Neural Network Classifiers on the PlantCLEF 2017 (right) and ExpertLifeCLEF 2018 (left) test sets.

		**PlantCLEF 2018—Accuracy [%]**		**PlantCLEF 2017—Accuracy [%]**	

**Architecture**	**Input**	**Images**	**Observations**	**Images**	**Observations**
ResNet-50	224 × 224	40.03	56.32	68.00	74.57
Inception-v4	224 × 224	43.41	59.41	71.32	77.92
Inception-Resnet-V2	224 × 224	44.14	68.15	70.57	78.96
ViT-Base/32	224 × 224	49.36	71.53	73.50	81.29
ViT-Base/16	224 × 224	51.58	73.70	75.54	82.57
EfficientNetV2-S	224 × 224	**53.08**	**74.13**	**79.21**	**85.75**
ViT-Tiny/16	384 × 384	47.43	69.06	73.64	80.59
SE-ResNeXt-101	384 × 384	54.61	73.75	80.31	85.98
ResNeSt-269e	384 × 384	56.27	74.52	81.68	86.74
ViT-Base/16	384 × 384	58.49	77.03	82.28	87.75
EfficientNetV2-L	384 × 384	59.90	77.03	84.15	88.52
ViT-Large/16	384 × 384	**67.03**	**83.54**	**86.87**	**91.15**

Observation values calculated as Mean Logits.

**Vision transformers:** The performance of different ViT architectures in the FGVC domain, multiple architectures, was evaluated for two different input resolutions—224 × 224 and 384 × 384—on two test sets—PlantCLEF2017 and ExpertLifeCLEF 2018. More precisely, ViT-Base/16 and ViT-Base/32 are compared on the input size of 224 × 224 and ViT-Large/16, ViT-Base/16 and ViT-Tiny/16 are tested on the input size of 384 × 384.

In the 384 × 384 scenario, ViT-Large/16 outperformed the best CNN model (ResNeSt-269e) 2.63% points on PlantCLEF 2017 and by 6.51% points on ExpertLifeCLEF 2018 while reducing the error by 22.91% and 28.34%, respectively. In the 224 × 224 scenario, the relative performance differed; EfficientNetV2-S outperformed all the models including both Vision Transformers on the ExpertLifeCLEF 2017 dataset. Comparison on the PlantCLEF2017 dataset, show the insignificant performance difference between ViT-Base/16 and EfficientNetV2-S.

### 5.2. Classification vs. metric learning

This section compares training a softmax image classifier explicitly as in the previous experiments and training an image retrieval system, which is subsequently used for nearest neighbor classification. The resolution of images, pre-trained weights and number of training epochs are kept the same across the two setups for a fair comparison. Even though we compare both methods under the same conditions, those conditions handicap the standard image classification approach as any additional techniques are permitted.

**Figure 4:**
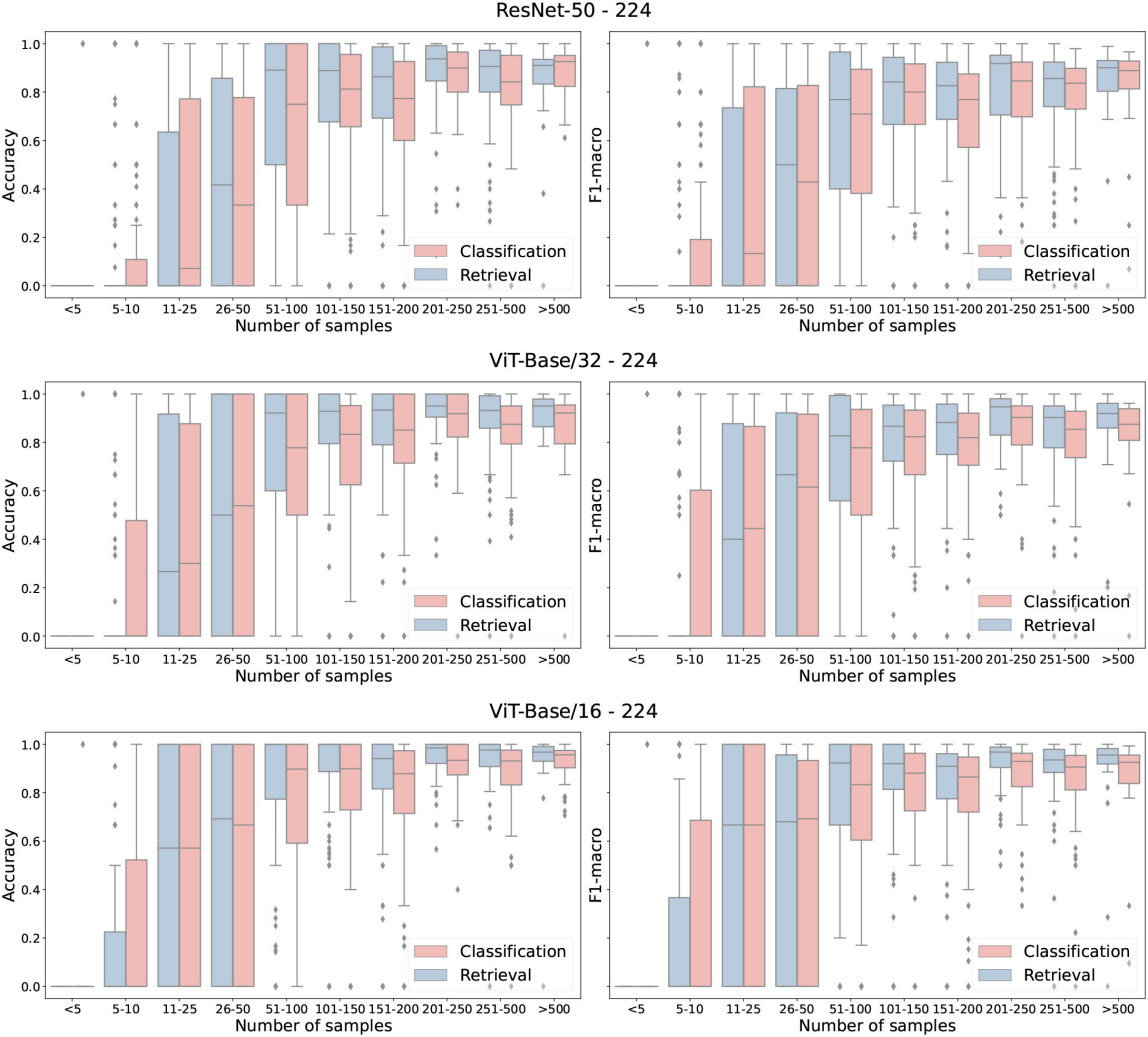
Classification performance (F1 and Accuracy) as box-plot for three backbone architectures and Classification and Retrieval approaches. Tested on PlantCLEF2017 test set with input resolution of 224 × 224.

**Figure 5:**
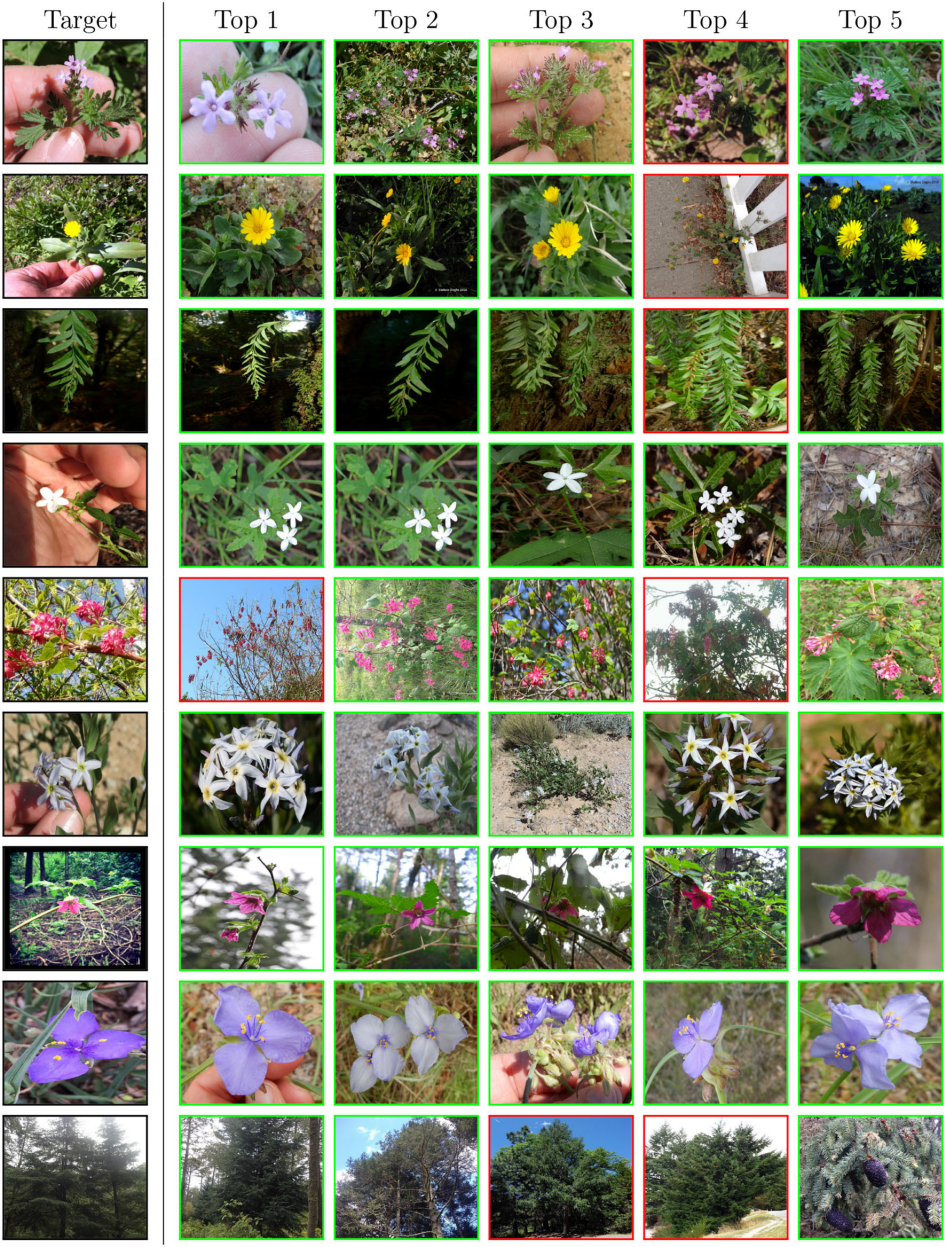
Qualitative examples from the retrieval approach on the iNaturalist dataset. The leftmost column shows samples from the test set followed by five nearest neighbors in the learned embedding space from the training set. The red box denotes the wrong species.

Overall, the retrieval approach achieved superior performance in all measured scenarios. Notably, the ViT-Base/16 feature extractor architecture achieved a higher classification accuracy with a margins of 0.28, 4.13, and 10.25% on ExpertLifeCLEF 2018, PlantCLEF 2017, and iNat2018–Plantae, respectively. Besides, the macro-F1 performance differences margin is noticeably higher—1.85% for ExpertLifeCLEF 2018 and 12.23% for iNat2018–Plantae datasets. Even though the standard classification approach performs better on classes with fewer samples (refer to Figure 4), common species with high a-prior probability are frequently wrongly predicted. This is primarily due to the high-class imbalance preserved in the dataset mimicked by the deep neural network optimized *via* SoftMax Cross-Entropy Loss. Thus, the results of the standard image classification approach performs way worst in case of the macro-F1 score. A full comparison of the classification and retrieval-based methods and their appropriate recognition scores are listed in Table 4. Three architectures—ResNet-50, ViT-Base/32, and ViT-Base/16 are evaluated. It can be seen from the results that for all selected architectures, retrieval leads to better performance. Furthermore, in Figure 5, we provide qualitative examples from the retrieval approach on the iNaturalist dataset. The Top5 predictions for randomly selected target images show that the retrieval-like approach allows better interpretability of the results.

**Table 4:** Performance evaluation for Classification (C) and Retrieval (R) based methods.

		**ExpertLifeCLEF 2018**		**PlantCLEF 2017**		**iNat2018–Plantae**	

**Architecture**	**Method**	**Acc**.	**Macro F1**	**Acc**	**Macro F1**	**Acc**	**Macro F1**
ResNet-50	C	59.87	55.11	77.89	54.48	57.73	52.69
ViT-Base/32	C	65.21	60.29	80.68	59.18	57.24	53.17
ViT-Base/16	C	71.71	67.35	84.48	65.40	67.42	64.51
ResNet-50	R	60.15	56.30	80.27	55.57	57.95	56.32
ViT-Base/32	R	66.48	61.49	84.89	60.79	63.12	61.24
ViT-Base/16	R	**71.99**	**69.20**	**88.61**	**66.39**	**77.67**	**76.74**

All models were trained for 100 epochs with fixed image size (224 × 224). No test-time augmentations were used. The most confident image prediction is used for all images belonging to the same observation.

### 5.3. A fine-tuning cookbook

In this section, we evaluate several methods that have the potential to increase performance for almost any deep neural network architecture considerably. The evaluation considers different loss functions, learning rate schedulers, prior estimation methods, and augmentations. Furthermore, the impact of the noisy data and the contribution of the test-time augmentations are studied. We list helpful methods and those that will make the performance worst if utilized. The evaluation is carried out on the PlantCLEF2017 and ExpertLifeCLEF 2018 datasets and ViT/Base-32 architecture with an input size of 224 × 224, if not stated differently. All used methods are described bellow. The ablation study for relevant methods is summarized in Table 5.

**Table 5:** Ablation study considering different techniques for ViT-Base/32 performance improvements.

			**Test 2018 - Acc [%]**		**Test 2017 - Acc [%]**	

**TTA**	**CCA**	**RC**	**Images**	**Observations**	**Images**	**Observations**
×	×	×	49.59	71.62	73.59	81.29
✓	×	×	+2.51	+1.98	+5.38	+4.65
×	✓	×	+0.32	+1.06	+0.70	+0.80
×	×	✓	–0.48	+1.30	+3.82	+3.86
×	✓	✓	–0.10	+1.93	+3.83	+3.89
✓	×	✓	+2.44	+2.51	+5.22	+4.22
✓	✓	×	**+3.01**	**+3.72**	+5.16	+4.38
✓	✓	✓	+2.83	+2.85	**+5.68**	**+4.67**

**Cyclic cosine annealing:** We compare standard cosine, a custom adaptive strategy where Learning Rate is decayed by 10% if validation loss is not reduced for two epochs, and Cyclic Cosine Annealing (CCA). The CCA is an alternative to standard Learning rate scheduling approaches, e.g., Exponential, Linear, Step, and Cosine. The CCA is divided into multiple cycles where the start learning rate decreases by 20%, and the learning rate in each cycle decreases *via* the standard cosine function. Such a learning rate schedule allows for diverging from local minima and searching for better optima. We compare standard cosine, a custom adaptive strategy where Learning Rate is decayed by 10% if validation loss is not reduced for two epochs, and Cyclic Cosine Annealing (CCA). Using the CCA instead of the standard approaches, we measured relative performance increases equal to +1.06 and +0.80% on the ExpertLifeCLEF 2018 and LifeCLEF2017, respectively.

**Test-time augmentations:** Test-time augmentations is a procedure where various mutations of the original image are feed-forwarded through the deep neural network in order to provide images in different rotations or scales. In our case, we use a simple test-time augmentation procedure—each test image is processed as a batch of 13 images:

- One original image (resized to 224 × 224 or 384 × 384),
- Four central crops covering 90, 80, and 70% of the original image size,
- Two top left corner crops covering 80 and 70% of the original image size,
- Two top right corner crops covering 80 and 70% of the original image size,
- Two bottom left corner crops covering 80 and 70% of the original image size,
- Two bottom right corner crops covering 80 and 70% of the original image size,

The predictions from all 13 cropped/augmented images are then combined. The results in Table 5 show than using so called test time augmentation improves the classification accuracy up to 1.98 and 4.65% on the ExpertLifeCLEF 2018 and LifeCLEF2017, respectively.

**Random crop:** Random crop allows for learning more detailed object representation as an image is not resized to a smaller resolution. Furthermore, training with random crops has high synergy with the test-time augmentation process if crops of similar size are used for TTA. For just a random crop, we measured performance increases equal to +1.30 and +3.86% achieved on the ExpertLifeCLEF 2018 and LifeCLEF2017, respectively. Combining with TTA, the margin increased to +1.93%, +3.89%.

**Prior shift adaptation:** The prior shift adaptation methods described in Sections 4.3.1 and 4.3.2 are compared in Table 6. Prior shift adaptation is applied to the prediction of each test augmentation, before the combination of augmentation and images per observation by averaging. The results show that in all cases, prior shift adaptation improves the recognition accuracy. The EM algorithm of Saerens et al. (2002) achieves the best result in three cases, the CM-L method of Sipka et al. (2022) in one case, but the differences are very small among the three compared prior shift adaptation methods.

**Table 6:** Accuracy before and after prior shift adaptation with the EM algorithm (Saerens et al., 2002) and the (S)CM-L methods (Sipka et al., 2022) on the ExpertLifeCLEF 2018 and the PlantCLEF 2017 test sets.

**Architecture**	**Test set**	**EM**	**CM-L**	**SCM-L**
ViT-Large/16	PlantCLEF 2017	+1.17	**+1.25**	+0.66
ViT-Large/16	ExpertLifeCLEF 2018	**+2.21**	+1.83	+1.64
SE-ResNeXt-101	PlantCLEF 2017	**+1.65**	+1.50	+1.07
SE-ResNeXt-101	ExpertLifeCLEF 2018	**+3.81**	+3.28	+3.23

All results are using the fine-tuned models and Mean Softmax Accuracy for combining predictions belonging to the same observation. Input size 384 × 384.

**Focal loss:** Even though commonly used in object detection, Focal Loss (Lin et al., 2017) has the potential to focus the training process on more challenging and rare samples and could prevent the vast majority of images from dominating the optimizer. As any considerable performance increase for ViT and CNN architectures was not measured on both datasets, we do not recommend using Focal Loss for plant recognition.

**Impact of the noisy data:** Noisy data, i.e., data without human-verified labels, are commonly used to increase the number of rare species samples and balance long-tailed class distribution. Even though the Krause et al. (2016) showed unreasonable effectiveness of the noisy labels on small-scale FGVC datasets, the contribution in the “in the wild” scenario is not established. In the case of the flora recognition, upsampling the minimum samples for each class (up to 10, 20, 30, and 40) did not improve the accuracy on both testing sets, i.e., the performance difference was statistically insignificant (see Table 7).

**Table 7:** Impact of additional noisy data on classification performance.

	**Test 2018 - Acc [%]**		**Test 2017 - Acc [%]**	

**Min. samples**	**Images**	**Observations**	**Images**	**Observations**
10	+0.17	–0.58	–0.20	–0.49
20	**+0.32**	–0.53	–0.33	–0.38
30	–0.13	–0.24	–0.44	–0.66
40	–0.10	–1.25	–0.60	–0.82
Baseline	49.77	**68.24**	**74.19**	**81.16**

## 6. Conclusion

The article assessed automatic plant identification as a fine-grained classification task on the largest available plant recognition datasets coming from the LifeCLEF and CVPR-FGVC workshops, counting up to 10,000 plant species.

**State-of-the-art classifiers:** The comparison of deep neural network classifiers in Section 5.1 shows the improvement in classification accuracy achieved by recent CNN architectures. The state-of-the-art Vision Transformers achieve even higher recognition scores: the best model, ViT-Large/16, achieves recognition scores of 91.15% and 83.54% on the PlantCLEF 2017 and ExpertLifeCLEF 2018 test sets, respectively, before additional post-processing like test-time augmentations and prior shift adaptation.

**Prior shift adaptation:** The prior shift in the datasets, i.e., the difference between the training and test data class distribution, is a significant and omnipresent phenomenon. We test existing prior shift adaptation methods and their impact on classification accuracy. The experiments with state-of-the-art methods for prior shift estimation (Saerens et al., 2002; Sipka et al., 2022), evaluated in Table 6, show that all three compared methods improve the classification accuracy in all cases. The differences among all three methods are rather small, EM achieving slightly better results in 3 of 4 cases. Given the optimization speed, EM algorithm is a preferred choice.

**Retrieval approach to fine-grained classification:** Training an image retrieval system and subsequently performing a nearest neighbor classification is a competitive alternative, with better results than direct classification. The prediction obtained *via* a nearest neighbor search is more interpretable as the samples contributing to the prediction can be visualized. Therefore, a retrieval-based approach is more suitable if utilized within the humans in the loop. On the other hand, the softmax predictions of a standard neural network classifier allow for simple post-processing procedures such as averaging and prior shift adaptation, which are yet to be explored for the retrieval approach, and which noticeably improve the final recognition accuracy of the standard classifiers.

Overall, using image-retrieval has clear advantages, e.g., recovering relevant nearest-neighbor labeled samples, providing ranked class predictions, and allows user or experts to visually verify the species based on the k-nearest neighbors Besides, the retrieval approach naturally supports open-set recognition problems, i.e., the ability to extend or modify the set of recognized classes after the training stage. The set of classes may change e.g., as a results of modifications to biological taxonomy. New classes are introduced simply by adding training images with the new label, whereas in the standard approach, the classification head needs re-training. On the negative side, the retrieval approach requires, on top of running the deep net to extract the embedding, to execute the nearest neighbor search efficiently, increasing the overall complexity of the fine-grained recognition system.

Contrary to our expectations, the error analysis in Figure 4 shows that the retrieval approach does not bring an improvement in classifying images from classes with few training samples. Figure 5 shows that retrieval has a very high accuracy for a higher number of species, but it also fails for a higher number of species.

## Data availability statement

The PlantCLEF datasets used in this study are publicly available in the repository of the LifeCLEF challenge organizers. The test set labels were kindly provided by the challenge Goëau et al. (2018) organizers. The iNaturalist dataset is publicly available at the competition GitHub page. All images used in the article are with CC-BY licence.

## Author contributions

LP, MŠ, YP, and JM conceived the study and drafted the manuscript. LP, MŠ, and YP implemented and conducted the machine learning experiments. All authors critically revised, reviewed, and approved the manuscript.

## Funding

LP was supported by the UWB project No. SGS-2022-017. LP and JM were supported by the Ministry of Environment of the Czech Republic project No. SS05010008. MŠ and JM were supported by Toyota Motor Europe. JM and YP were supported by Research Center for Informatics (project CZ.02.1.01/0.0/0.0/16\_019/0000765 funded by OP VVV). YP was supported by the Grant Agency of the Czech Technical University in Prague, grant No. SGS20/171/OHK3/3T/13, by Project StratDL in the realm of COMET K1 center Software Competence Center Hagenberg and an Amazon Research Award.

## Conflict of interest

The authors declare that the research was conducted in the absence of any commercial or financial relationships that could be construed as a potential conflict of interest.

## Publisher's note

All claims expressed in this article are solely those of the authors and do not necessarily represent those of their affiliated organizations, or those of the publisher, the editors and the reviewers. Any product that may be evaluated in this article, or claim that may be made by its manufacturer, is not guaranteed or endorsed by the publisher.

## Footnotes

^1^We use the term class following the machine learning wording, where classes denote the categories to be recognized, not the taxonomic rank (*classis*), i.e., we use the term class for species.
